# Cost-effectiveness of low-dose aspirin for the prevention of preterm birth: a prospective study of the Global Network for Women’s and Children’s Health Research

**DOI:** 10.1016/S2214-109X(22)00548-4

**Published:** 2023-03

**Authors:** Jackie K Patterson, Simon Neuwahl, Norman Goco, Janet Moore, Shivaprasad S Goudar, Richard J Derman, Matthew Hoffman, Mrityunjay Metgud, Manjunath Somannavar, Avinash Kavi, Jean Okitawutshu, Adrien Lokangaka, Antoinette Tshefu, Carl L Bose, Abigail Mwapule, Musaku Mwenechanya, Elwyn Chomba, Waldemar A Carlo, Javier Chicuy, Lester Figueroa, Nancy F Krebs, Saleem Jessani, Sarah Saleem, Robert L Goldenberg, Kunal Kurhe, Prabir Das, Archana Patel, Patricia L Hibberd, Emmah Achieng, Paul Nyongesa, Fabian Esamai, Sherri Bucher, Edward A Liechty, Brian W Bresnahan, Marion Koso-Thomas, Elizabeth M McClure

**Affiliations:** Department of Pediatrics, University of North Carolina at Chapel Hill, Chapel Hill, NC, USA; RTI International, Research Triangle Park, NC, USA; RTI International, Research Triangle Park, NC, USA; RTI International, Research Triangle Park, NC, USA; Jawaharlal Nehru Medical College, KLE University, Belagavi, India; Department of Obstetrics and Gynecology, Thomas Jefferson University, Philadelphia, PA, USA; Department of Obstetrics and Gynecology, Christiana Care, Newark, DE, USA; Jawaharlal Nehru Medical College, KLE University, Belagavi, India; Jawaharlal Nehru Medical College, KLE University, Belagavi, India; Jawaharlal Nehru Medical College, KLE University, Belagavi, India; Kinshasa School of Public Health, University of Kinshasa, Kinshasa, Democratic Republic of the Congo; Kinshasa School of Public Health, University of Kinshasa, Kinshasa, Democratic Republic of the Congo; Kinshasa School of Public Health, University of Kinshasa, Kinshasa, Democratic Republic of the Congo; Department of Pediatrics, University of North Carolina at Chapel Hill, Chapel Hill, NC, USA; University Teaching Hospital, Lusaka, Zambia; University Teaching Hospital, Lusaka, Zambia; University Teaching Hospital, Lusaka, Zambia; Department of Pediatrics, University of Alabama at Birmingham, Birmingham, AL, USA; Instituto de Nutrición de Centro América y Panamá, Guatemala City, Guatemala; Instituto de Nutrición de Centro América y Panamá, Guatemala City, Guatemala; School of Medicine, University of Colorado, Aurora, CO, USA; Department of Community Health Sciences, Aga Khan University, Karachi, Pakistan; Department of Community Health Sciences, Aga Khan University, Karachi, Pakistan; Department of Obstetrics and Gynecology, Columbia University, New York, NY, USA; Lata Medical Research Foundation, Nagpur & Datta Meghe Institute of Medical Sciences, Sawangi, India; Lata Medical Research Foundation, Nagpur & Datta Meghe Institute of Medical Sciences, Sawangi, India; Lata Medical Research Foundation, Nagpur & Datta Meghe Institute of Medical Sciences, Sawangi, India; School of Public Health, Boston University, Boston, MA, USA; Department of Child Health and Paediatrics, School of Medicine, Moi University, Eldoret, Kenya; Department of Child Health and Paediatrics, School of Medicine, Moi University, Eldoret, Kenya; Department of Child Health and Paediatrics, School of Medicine, Moi University, Eldoret, Kenya; School of Medicine, Indiana University, Indianapolis, IN, USA; School of Medicine, Indiana University, Indianapolis, IN, USA; Department of Radiology, University of Washington, Seattle, WA, USA; Eunice Kennedy Shriver National Institute of Child Health and Human Development, National Institutes of Health, Bethesda, MD, USA; RTI International, Research Triangle Park, NC, USA

## Abstract

**Background:**

Premature birth is associated with an increased risk of mortality and morbidity, and strategies to prevent preterm birth are few in number and resource intensive. In 2020, the ASPIRIN trial showed the efficacy of low-dose aspirin (LDA) in nulliparous, singleton pregnancies for the prevention of preterm birth. We sought to investigate the cost-effectiveness of this therapy in low-income and middle-income countries.

**Methods:**

In this post-hoc, prospective, cost-effectiveness study, we constructed a probabilistic decision tree model to compare the benefits and costs of LDA treatment compared with standard care using primary data and published results from the ASPIRIN trial. In this analysis from a health-care sector perspective, we considered the costs and effects of LDA treatment, pregnancy outcomes, and neonatal health-care use. We did sensitivity analyses to understand the effect of the price of the LDA regimen, and the effectiveness of LDA in reducing both preterm birth and perinatal death.

**Findings:**

In model simulations, LDA was associated with 141 averted preterm births, 74 averted perinatal deaths, and 31 averted hospitalisations per 10 000 pregnancies. The reduction in hospitalisation resulted in a cost of US$248 per averted preterm birth, $471 per averted perinatal death, and $15·95 per disability-adjusted life year.

**Interpretation:**

LDA treatment in nulliparous, singleton pregnancies is a low-cost, effective treatment to reduce preterm birth and perinatal death. The low cost per disability-adjusted life year averted strengthens the evidence in support of prioritising the implementation of LDA in publicly funded health care in low-income and middle-income countries.

**Funding:**

Eunice Kennedy Shriver National Institute of Child Health and Human Development.

## Introduction

Globally, an estimated 15 million pregnancies result in live preterm births each year.^1^ More than 1 million of these infants born prematurely die before they are 5 years old, making prematurity the leading cause of death for children younger than 5 years.^2^ Rates of preterm birth are increasing worldwide, and low-income and middle-income countries (LMICs) disproportionately share the burden of death due to prematurity.^3^ In addition to an increased risk of mortality, premature infants are at significant risk for morbidity, including prolonged birth hospitalisation and neurodevelopmental impairment. Strategies to prevent preterm birth are few in number and largely expensive. As such, low-cost, effective strategies to prevent preterm birth are an urgent and unmet public health need.

In 2020, the National Institute of Child Health and Human Development Global Network for Women’s and Children’s Health Research evaluated the efficacy of low-dose aspirin (LDA [81 mg aspirin]) for the prevention of preterm birth in the ASPIRIN trial.^4^ This trial showed that once-a-day LDA for nulliparous women initiated between 6 and 13 weeks’ gestation and continued until 36 weeks’ gestation reduces preterm birth and perinatal mortality (defined as death between 20 weeks’ gestation and within 7 days after birth).^4^ In this multinational randomised trial in LMICs, there was no change in maternal hypertensive disorders, haemorrhage, or maternal mortality in women who received LDA. Additionally, there was no increase in serious adverse events in pregnant women taking LDA nor in their fetuses, suggesting that LDA is a safe therapy for this population.^5^ Given the diverse group of 11 976 women from six countries enrolled in the study, the ASPIRIN trial^4^ showed the benefit of LDA for the prevention of preterm birth and perinatal mortality. The tolerability and low cost of LDA make it a promising therapy to implement in LMICs, in which the burden of mortality from prematurity is highest.

To support policy makers considering recommendations regarding LDA in nulliparous, singleton pregnancies in LMICs, we sought to determine the cost-effectiveness of this intervention. We aimed to estimate the incremental cost per preterm birth, perinatal death, and disability-adjusted life year (DALY) averted.

## Methods

### Study design and participants

This was a cost-effectiveness analysis of LDA for the prevention of preterm birth. Our analysis reflected the health-care sector perspective—including only direct medical costs without additional societal costs, such as lost wages, and no longer-term medical costs beyond the neonatal period for survivors. This focus was driven by scarcity of data in LMICs to support a rigorous analysis from a societal perspective.

In this study, we used data from the ASPIRIN trial^4^ to estimate the cost-effectiveness of LDA to prevent preterm birth and perinatal death. The ASPIRIN trial^4^ assessed once-a-day LDA for the prevention of preterm birth in nulliparous women (ie, women who have never given birth to a liveborn neonate, but might have had a previous miscarriage, elective abortion, or stillbirth) in a randomised, double-blind, placebo-controlled trial. Nulliparous women with a singleton pregnancy, confirmed with ultrasound, between 6 weeks and 0 days and 13 weeks and 6 days gestation, were eligible to participate. Women with an aspirin allergy, those who had previously received aspirin therapy for more than 1 week during the current pregnancy, those with a history of more than two pregnancy losses in the first trimester, or those with a medical condition for which LDA was indicated were excluded. Participants were enrolled in the trial from March 23, 2016, to April 11, 2019, at seven sites in six countries (one low-income country [LIC]: DR Congo; four lower-middle-income countries: India [two sites], Kenya, Pakistan, and Zambia; and one upper-middle-income country: Guatemala). These sites were a mix of primary, secondary, and tertiary care facilities.

The relevant ethics committees and regulatory agencies of each participating site and the ethics committees of the US-based partners and Research Triangle Institute International approved the ASPIRIN trial protocol (appendix p 1). All women provided informed consent before participation in the original trial. No permissions were required for this cost-effectiveness analysis.

### Procedures

In the original ASPIRIN trial,^4^ women were randomly assigned (1:1) to receive once-a-day oral LDA (81 mg) or placebo tablets of identical appearance until 36 weeks and 0 days of pregnancy. Details of the study methods and results have been previously published.^4,6^ In this cost-effectiveness analysis, we draw on published data from the ASPIRIN trial^4^ and from a cost analysis for country-specific health-care use costs (ie, hospitalisation and associated therapies) relevant for five of the study sites from the ASPIRIN trial (all sites except India).^7^

**Figure 1:**
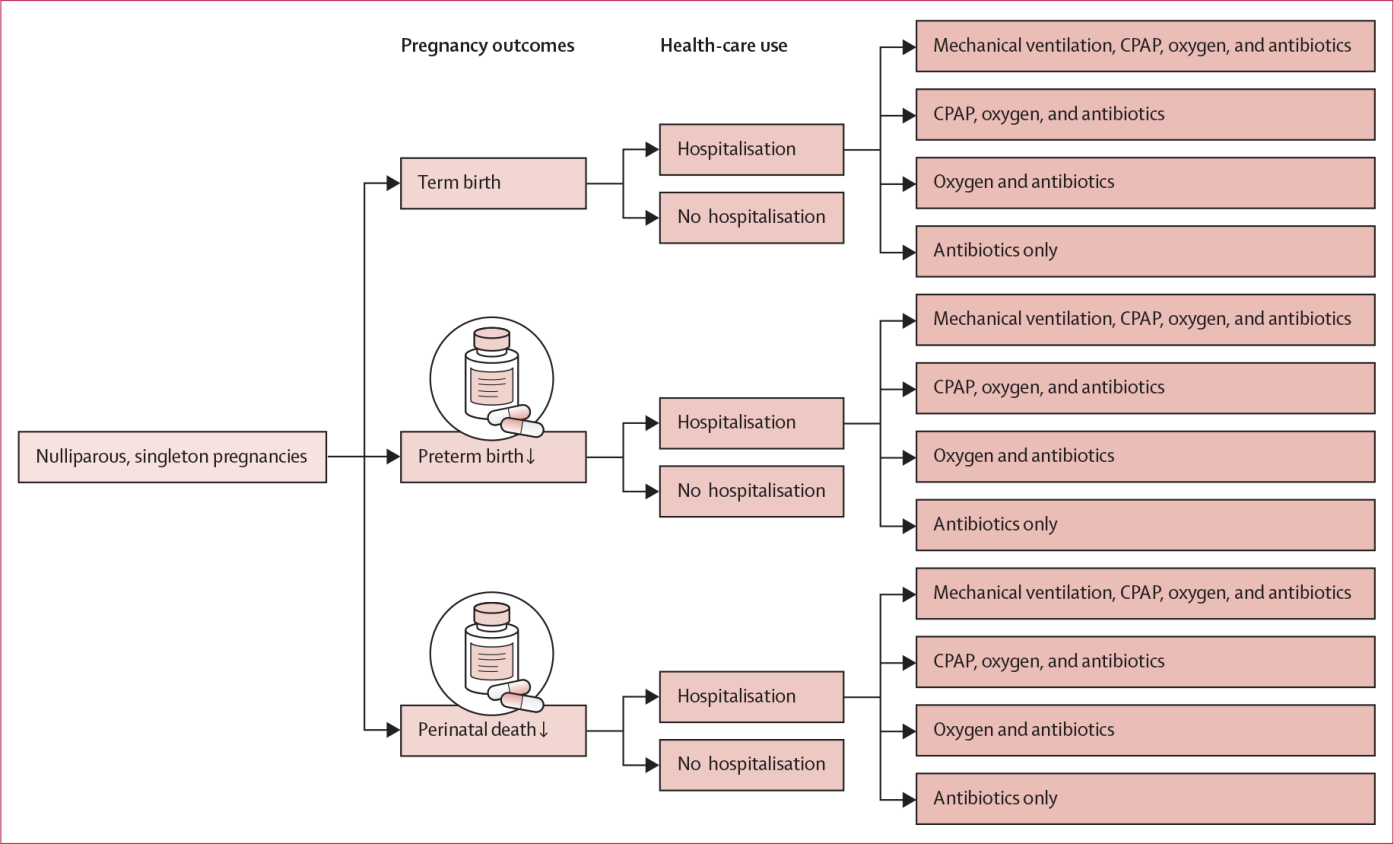
Model design We used this model to compare costs and benefits between standard care and LDA treatment. The first branching of the model separates pregnancy outcomes into mutually exclusive branches of term birth, preterm birth, and perinatal death. Term or preterm infants who died within 7 days of birth were not included in the term or preterm branches. When we ran this model for our LDA-treated sample, we reduced preterm birth and perinatal death per the relative risks reported in the ASPIRIN trial.^4^ Subsequent branches account for infant health-care use with hospitalisations and associated therapies, assuming receipt of a given therapy included receipt of all lower-intensity therapies. All branches are mutually exclusive, resulting in 15 different scenarios. CPAP=continuous positive airway pressure. LDA=low-dose aspirin.

For this cost-effectiveness analysis, we developed a model to compare costs and health outcomes between standard care and LDA treatment that accounted for pregnancy outcomes and health-care use, resulting in 15 mutually exclusive scenarios (figure 1). Although we considered both maternal and neonatal health-care use, the final model includes only neonatal health-care use due to an absence of difference in maternal secondary outcomes, medication side-effects, and antenatal health-care use between the placebo and intervention groups in the ASPIRIN trial.^4^ We rank-ordered neonatal therapies associated with hospitalisation by intensity (mechanical ventilation being most intense followed by continuous positive airway pressure, oxygen, and antibiotics), and assumed that receipt of a given therapy included receipt of all lower-intensity therapies. This resulted in a simplified set of mutually exclusive branches intended to avoid unnecessary model complexity from combinations of therapies rarely observed in the trial data (eg, mechanical ventilation without oxygen).

We used the same estimates for treatment costs, pregnancy outcomes, treatment effects, health-care use, and disability weight across all cost-effectiveness analyses (table 1). We calculated the cost of LDA tablets assuming a 217-day supply (reflecting initiation of therapy at 6 weeks and 0 days and continuation until 36 weeks and 0 days of pregnancy) using the median cost from the 2015 International Medical Products Price Guide.^8^ We assumed LDA would be dispensed at regular antenatal care visits; therefore, we did not include the costs of visits for pill counting and recording of adverse events that were part of the ASPIRIN trial.^6^ We established all baseline pregnancy outcome probabilities using data from the placebo group of the ASPIRIN trial,^4^ and adjusted these probabilities for treatment effect in the LDA-treated sample using the relative risks reported in the ASPIRIN trial.^4^ We assumed that differences in neonatal health-care use between the placebo and intervention groups in the ASPIRIN trial were mediated by the effect of LDA on prematurity or perinatal death, and thus used data from all participants to calculate health-care use probabilities. We stratified these probabilities by mutually exclusive categories of term birth, preterm birth, and perinatal death to account for the higher hospitalisation rate observed in each group (table 1).

We evaluated costs for individual countries using country-specific data for health-care use costs, life expectancy, and gross domestic product (GDP) per capita (table 2). Due to regional similarities in health-care costs and life expectancy, we grouped Kenya and Zambia in our analysis. Country-specific health-care use costs were derived from the works published by Bresnahan and colleagues^7,9^ that reported costs from 2015.^7,9^ We inflated these costs reported in 2015 to the value of the US$ in 2020 using the latest GDP price deflator data available from the World Bank.^10^ Local health researchers at the Belagavi, India, site estimated costs for India using private hospital data from 2021; we converted these costs to US$ using a conversion rate of 0·013 rupees=$1·00. Due to different health-care finance systems, labour costs, medical supplies costs, and other factors, costs by service are not expected to be consistent. We extrapolated all life expectancy data from the 2019 WHO Global Health Observatory, using the data on both sexes combined.^11^ We identified GDP per capita in US$ using World Bank data.^12^

### Outcomes

The primary outcome of the original study^4^ was preterm birth (defined as birth before 37 weeks’ gestational age), analysed in women with pregnancy outcomes at or after 20 weeks’ gestation. Our model presents key results of preterm births and perinatal deaths averted by LDA treatment, expressed as cost-effectiveness results by calculating a cost per preterm birth averted and a cost per perinatal death averted. To determine DALYs averted, we estimated years of life saved with each perinatal death averted using country-specific life expectancy data (table 2). We also incorporated a disability effect for preterm birth (0·001), based on the Institute for Health Metrics and Evaluation (IHME) Global Burden of Disease (GBD), University of Washington, Seattle, WA, USA, years of life with disability estimate.^13^ Estimates from IHME GBD 2019 show the biggest effect of preterm birth occurs via infant mortality.^13^ The perinatal death branch of our model captures the effect of preterm birth on mortality in the first week after birth. Although as many as 20% of infant deaths attributable to prematurity occur between 1 week and 1 year of life,^14^ we did not capture the effect of reductions in preterm birth on infant mortality beyond 1 week. We divided incremental cost by DALYs averted. The cost per DALY is reported to facilitate comparisons of cost-effectiveness across interventions that affect all types of health outcomes. It is also reported alongside a country’s GDP to provide country-specific context for the cost-effectiveness of LDA. We discounted future life years saved due to perinatal deaths averted back to the present using a discount rate of 3%.^15^ All model costs were accumulated in the first year and were not discounted. We report model costs according to the 2020 value of the US$, except for India which we report in 2021 US$.

### Statistical analysis

To analyse the cost-effectiveness of LDA for the prevention of preterm birth and perinatal mortality, we developed a probabilistic decision tree model using TreeAge Pro 2019 (version 2.1). We assessed the incremental cost-effectiveness of an LDA-treated model sample compared with a standard care model sample using primary data from the ASPIRIN trial.^4^ We defined incremental cost-effectiveness as the incremental costs divided by the DALYs averted (per 10 000 nulliparous, singleton pregnancies).

After finalising the model design, SN and JM internally validated the data by removing model treatment effects and comparing model branch and sub-branch results that should be identical in their expected values. We reconciled all discrepancies and checked expected values in each branch again to confirm that the model was calculating outcomes as expected.

We ran the model to compare standard care with LDA treatment by defining all key parameters in table 1 as a distribution and generating 10 000 randomly drawn parameter sets to use in model calculation. Our results represent the mean of all 10 000 simulations of the model, with each simulation using a different parameter set (drawn from the same distribution). We used the country-specific health-care use costs and life expectancy data presented in table 2.

To incorporate a wider range of uncertainty in our analysis, we ran detailed sensitivity analyses by changing the point estimates for three key parameters: the price of the LDA regimen, the effectiveness of LDA in reducing preterm birth, and the effectiveness of LDA in reducing perinatal death. Low LDA cost and high LDA cost estimates were based on the low ($0·0042 per day) and the high ($0·0393 per day) price per aspirin tablet from the 2015 International Medical Products Price Guide.^8^ We derived the low and high LDA effectiveness estimates from the upper and lower 95% CI values published in the efficacy data from the ASPIRIN trial.^4^ We also investigated the effect of other model parameters on incremental cost using a Tornado diagram.

### Role of the funding source

Staff from the funder participated in data interpretation, and reviewed and approved the manuscript. The funder had no role in data collection or analyses.

## Results

As previously published, the ASPIRIN trial enrolled 11 976 nulliparous women (mean maternal age 20·9 years [SD 3·3]) who were randomly assigned to receive daily LDA or placebo.^4^ Baseline characteristics, including maternal age, previous pregnancies, fetal gestational age at enrolment, level of education, anthropometry, and antenatal care visits were similar between groups; delivery characteristics, such as delivery attendant, location, and mode, were also similar between groups.^4^ Daily LDA reduced preterm delivery before 37 weeks (relative risk 0·89 [95% CI 0·81–0·98]) and perinatal mortality (0·86 [0·73–1·00]).^4^

In this cost-effectiveness analysis, LDA was associated with 140·9 (95% CI 85·8–185·8) preterm births averted, 74·1 (43·6–95·9) perinatal deaths averted, and 31·8 (30·7–30·9) neonatal hospitalisations averted per 10 000 pregnancies in each country (table 3). Cost-related and DALY-related results in table 3 incorporate country-specific parameters. Pakistan represented the median country in terms of life expectancy, GDP per capita, and cost-effectiveness of LDA. The total cost of the LDA intervention in Pakistan was $38 470·02 per 10 000 pregnancies. The reduction in hospitalisations and hospital-based procedures lowered overall costs by $3545·15, resulting in an incremental cost of $34 925·93 per 10 000 pregnancies. This yielded cost-effective ness results of $248·96 per preterm birth averted, $471·32 per perinatal death averted, and $15·95 per DALY averted (table 3).

Country-specific analyses showed a range of incremental costs from $29 450·03 in Guatemala to $62 745·42 in DR Congo (table 3). Incremental costs were lowest in Guatemala due to the high cost of hospitalisation; therefore, the higher cost-savings were from reduced hospitalisations. Incremental costs were the highest in DR Congo due to the small cost-savings associated with reduced hospitalisations in this low-income country and a very high rate of inflation from 2015 to 2020 with prices more than doubling. Guatemala had the lowest cost per preterm birth averted ($208·81) and per perinatal death averted ($397·48); DR Congo had the highest ($445·10) cost per preterm birth averted and per perinatal death averted ($847·13; table 3). DALYs averted ranged from 2155 years in DR Congo to 2251 years in Guatemala, and resulted in a range in cost per DALY of $13·08 to $29·12. Guatemala had the lowest cost per DALY averted ($13·08) due to the higher cost of hospitalisation and associated therapies (resulting in lower incremental costs for LDA treatment) and the longer life expectancies (resulting in more DALYs averted). Due to lower costs of hospitalisation, shorter life expectancy, and higher inflation, LDA treatment was less cost-effective in DR Congo ($29·12 per DALY averted). The cost per DALY averted represented about 5% or less of each country’s GDP per capita (table 3).

Using the results from Pakistan as our base case, we did several one-way sensitivity analyses (table 4). The incremental cost of the LDA regimen yielded a cost per preterm birth averted that ranged from $40·36 to $568·24 (compared with the base case cost of $247·96; table 4). The cost per perinatal death averted ranged from $76·72 to $1090·34 (base case cost $471·32), and cost per DALY averted ranged from $2·60 to $36·89 (base case cost $15·95). One-way sensitivity analysis on the effectiveness of LDA in reducing preterm birth yielded a range of 26 to 249 preterm births averted per 10 000 pregnancies (compared with the base case of 141 preterm births averted per 10 000 pregnancies; table 4). The low LDA effectiveness scenario resulted in a cost per preterm birth averted of $1393·98 compared with $134·65 in the high LDA effectiveness scenario (base case cost per preterm birth averted $247·96). Varying the effectiveness of LDA on preterm birth resulted in a cost per DALY averted ranging from $15·28 to $16·65. Varying effectiveness of LDA on perinatal death resulted in two preterm births averted per 10 000 pregnancies in the low LDA effectiveness case, increasing the cost per DALY averted from $15·95 to $766·14. With high LDA effectiveness in reducing perinatal death, there were 139 perinatal deaths averted per 10 000 pregnancies, decreasing the cost per DALY averted from $15·95 to $8·13.

**Figure 2:**
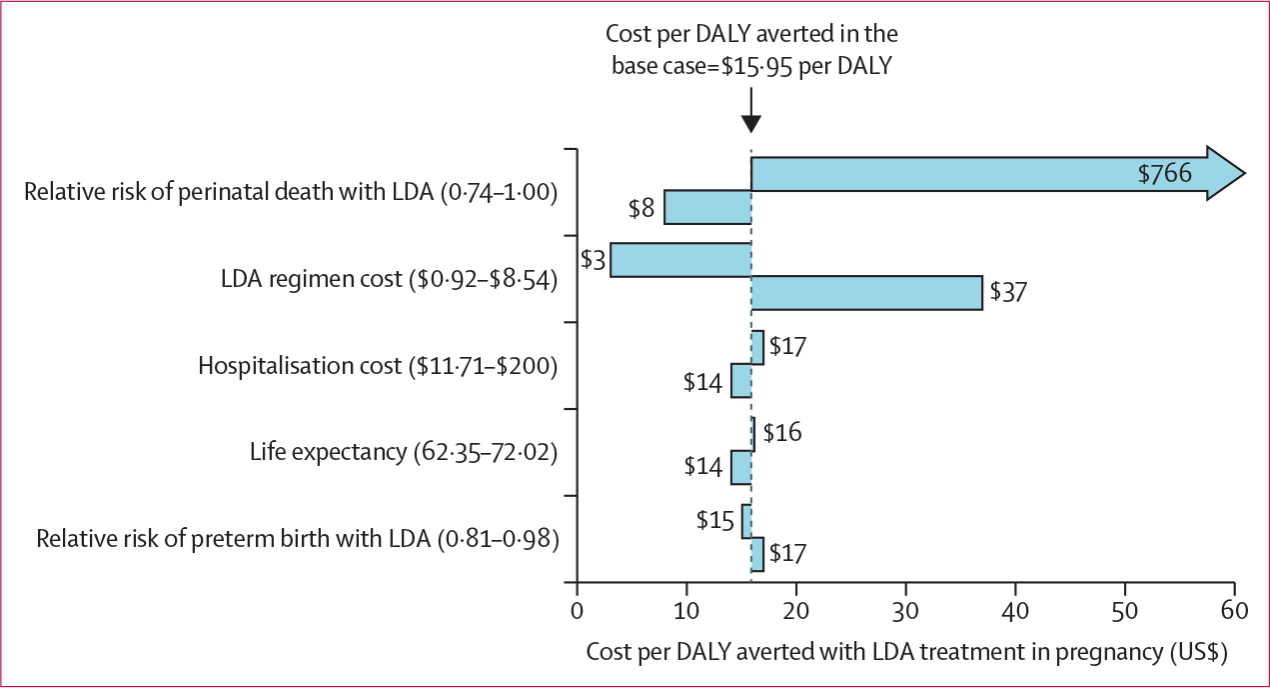
Cost per DALY averted in US$ The cost per DALY averted for the base case reflects health-care use cost data in Pakistan, which represented the median cost of the ASPIRIN trial sites. Results are sorted by their effect on the cost per DALY averted with LDA treatment. The low and high LDA cost were based on the low ($0·0042 per day) and the high ($0·0393 per day) price per tablet in the 2015 International Medical Products Price Guide (inflated to 2020 US$ using the gross domestic product deflator for Pakistan). Low and high LDA effectiveness estimates were based on the upper and lower 95% CI values reported in the ASPIRIN trial.^4^ For hospitalisation costs, the highest cost was in Pakistan ($94·00), but we increased the high cost to $200·00 to explore the effect of a higher than expected cost. Finally, we varied life expectancy across the range represented by the ASPIRIN trial sites (DR Congo had the shortest life expectancy [62·35 years] and Guatemala had the longest [72·02 years]). DALY=disability-adjusted life year. LDA=low-dose aspirin.

The relative risk of perinatal death with LDA and the cost of LDA had the largest effect on the cost per DALY averted (figure 2). Meanwhile, hospitalisation costs, life expectancy, and the relative risk of preterm birth each had a modest effect on the cost per DALY averted over a feasible range of values defined for each. In particular, our sensitivity analysis (figure 2) tested the sensitivity of the model to higher hospitalisation costs ($200·00), showing that cost-effectiveness only improves from $15·95 per DALY averted to $14·39. For LDA treatment to become cost-saving (ie, negative incremental costs), the cost of hospitalisation would have to be $3500 or more per hospitalisation, assuming all other parameters were unchanged.

## Discussion

Using data from the large-scale, multinational ASPIRIN trial,^4^ we found that LDA treatment for nulliparous, singleton pregnancies is an affordable and probably highly cost-effective intervention that reduces preterm birth and perinatal death. The cost-effectiveness of LDA treatment varied only modestly by country, but was very sensitive to the price of the LDA regimen and the effectiveness of LDA in reducing perinatal death. Because of the modest reduction in hospitalisations attributable to LDA and the low cost of hospitalisation in LMICs, cost-effectiveness of LDA treatment is not very sensitive to health-care use costs.

The cost-effectiveness of LDA for nulliparous, singleton pregnancies at $15·95 per DALY averted compares favourably with other treatments for mothers and newborn babies commonly implemented in LMICs.^16^ For example, early newborn care training of clinic midwives in Zambia costs $5·24, training traditional birth attendants and supplying them with clean delivery kits costs $74,^17,18^ distributing insecticide-treated bednets for malaria prevention during antenatal care in DR Congo costs $17·22,^19^ and providing maternal and neonatal home care in Bangladesh costs $103·49 per DALY averted.^20^

The World Bank recommends implementation of interventions costing less than $200·00 per DALY averted in LICs and less than $500·00 per DALY averted in lower-middle income countries.^21^ Given the LDA treatment cost per DALY averted of $15·95, our cost-effectiveness assessment (which incorporates high-quality evidence from a well controlled clinical trial) supports the use of LDA for nulliparous, singleton pregnancies. This study can inform public-funding decisions for health-care interventions in countries of all income classifications, including LICs, lower-middle-income countries, and upper-middle-income countries which were all represented in the ASPIRIN trial.^4^ The cost per DALY averted in this analysis of the ASPIRIN trial was well below the per capita GDP in each country, one measure of the willingness-to-pay threshold for decision making.^22^

An important strength of our modelling analysis is the volume and specificity of data used directly from the ASPIRIN trial.^4^ We used published results from the ASPIRIN trial to define probabilities for pregnancy outcomes and to estimate treatment effect. We also used primary data from the ASPIRIN trial^4^ to generate probabilities for hospitalisation and the use of associated therapies. Health-care use costs for all sites, other than India, were derived from a previously published analysis of costs in the same study sites of the ASPIRIN trial.^7^

Although a strength of our analysis was the use of data from the ASPIRIN trial, the data reflects efficacy rather than effectiveness. Although women were enrolled at a median gestational age of 10 weeks in the ASPIRIN trial, women in LMICs typically present for antenatal care much later. Furthermore, medication compliance in the real-world setting might be reduced compared with the compliance achieved in the trial. Given these considerations, our sensitivity analysis reflecting low LDA effectiveness might be a better approximation of real-world implementation. Implementation strategies that promote early antenatal care and high compliance with LDA will be crucial to achieve the benefit of LDA in scale-up.

There are several limitations to consider for this analysis. In keeping with the original trial,^4^ we focused on all preterm neonates rather than calculating separate probabilities and costs for early preterm versus late preterm neonates. As such, our analysis does not account for differential health-care use based on degree of prematurity. Per our sensitivity analysis, this is unlikely to have a substantial effect on the cost-effectiveness of LDA treatment given the modest reduction in hospitalisations and low costs of hospitalisation observed in this analysis. In keeping with the original trial,^4^ we report the effect of averting preterm birth on mortality up to the first week after birth, but we do not capture its effect on infant mortality beyond the first week. We did not account for future medical costs for neonates saved by the intervention given the limited country-specific data on future lifetime costs for neonates in LMICs. We did not estimate out-of-pocket expenses for hospital care nor the costs of broader implementation of the intervention, including infrastructure development to support early attendance at antenatal care visits. Although we show country-specific cost-effectiveness using local cost data, effectiveness reflects combined rather than country-specific estimates based on the data reported in the primary trial. When the effect on perinatal death is removed per our one-way sensitivity analysis, the cost per DALY averted of $766·14 is higher than the upper limits set by the World Bank for implementation of interventions in both LICs and lower-middle-income countries. Nevertheless, our main results incorporate the uncertainty of the perinatal death effect using probabilistic sensitivity analysis of more than 10 000 simulations. Our base case results remained robust to this uncertainty, with a 95% CI upper limit of $27·11 per DALY averted, which is well below the recommended limit of $200·00 per DALY averted for implementation in LICs.

In conclusion, LDA treatment in nulliparous, singleton pregnancies is a low-cost, effective treatment to reduce preterm birth and perinatal death. Relatively low cost per DALY averted estimates strengthen the evidence for policy makers’ decisions related to implementing LDA treatment in publicly funded health care in LMICs.

## Supplementary Material

MMC1

## Figures and Tables

**Table 1:** Parameters for all cost-effectiveness analyses

	Mean (SD)	Distribution	Source

**Treatment costs**			
LDA regimen cost in 2020, US$*	$3.83 (0.74)	Uniform	2015 International Medical Products Price Guide
**Pregnancy outcome probabilities**			
Preterm birth	0.131 (0.004)	Beta	ASPIRIN trial results†
Perinatal death	0.054 (0.003)	Beta	ASPIRIN trial results†
**Treatment effects**			
Relative risk of preterm birth	0.89 (0.04)	Log-normal	ASPIRIN trial results†
Relative risk of perinatal death	0.86 (0.07)	Log-normal	ASPIRIN trial results†
**Health-care use probabilities for term birth**			
Hospital admission	0.078 (0.004)	Beta	ASPIRIN trial data‡
**Therapies associated with hospitalisation**			
Antibiotics	0.190 (0.028)	Beta	ASPIRIN trial data‡
Antibiotics and oxygen	0.620 (0.022)	Beta	ASPIRIN trial data‡
Antibiotics, oxygen, and CPAP	0.072 (0.013)	Beta	ASPIRIN trial data‡
Antibiotics, oxygen, CPAP, and mechanical ventilation	0.118 (0.021)	Beta	ASPIRIN trial data‡
**Health-care use probabilities for preterm birth**			
Hospitalisation	0.197 (0.015)	Beta	ASPIRIN trial data‡
**Therapies associated with hospitalisation**			
Antibiotics	0.170 (0.024)	Beta	ASPIRIN trial data‡
Antibiotics and oxygen	0.599 (0.041)	Beta	ASPIRIN trial data‡
Antibiotics, oxygen, and CPAP	0.088 (0.020)	Beta	ASPIRIN trial data‡
Antibiotics, oxygen, CPAP, and mechanical ventilation	0.143 (0.032)	Beta	ASPIRIN trial data‡
**Health-care use probabilities for perinatal death**			
Hospitalisation	0.267 (0.030)	Beta	ASPIRIN trial data‡
**Therapies associated with hospitalisation**			
Antibiotics	0.087 (0.011)	Beta	ASPIRIN trial data*
Antibiotics and oxygen	0.543 (0.063)	Beta	ASPIRIN trial data‡
Antibiotics, oxygen, and CPAP	0.074 (0.031)	Beta	ASPIRIN trial data‡
Antibiotics, oxygen, CPAP, and mechanical ventilation	0.296 (0.051)	Beta	ASPIRIN trial data‡
**Disability weight**			
Preterm birth	0.001 (0.00)	Uniform	IHME GBD, 2019

CPAP=continuous positive airway pressure. GBD=Global Burden of Disease. IHME=Institute for Health Metrics and Evaluation. LDA=low-dose aspirin.

* The cost of the LDA regimen is based on a 217-day supply of once-a-day 81 mg tablets with enteric coating using the median cost of $0⋅0177 per day derived from the 2015 International Medical Products Price Guide and inflated to 2020 using the gross domestic product price deflator from the World Bank for the median country (Pakistan). 217 days reflects initiation of therapy at 6 weeks and 0 days and continuation until 36 weeks and 0 days of pregnancy.

† Values derived from published ASPIRIN trial results.^4^

‡ Values derived from unpublished calculations using primary ASPIRIN trial data.

**Table 2:** Cost and life expectancy parameters for country-specific analyses

	DR Congo	Guatemala	India	Kenya and Zambia*	Pakistan

Health-care use costs, US$					
Hospitalisation cost per admission	$11.71	$87.27	$18.20	$68.29	$94.00
Therapies associated with hospitalisation, US$					
Antibiotics	15.06	13.49	0.78	54.53	12.28
Oxygen	NA†	6.93	69.29	2.73	5.97
CPAP	NA†	6.93	112.58	2.73	5.97
Mechanical ventilation	NA†	44.51	112.58	1.95	5.97
Life expectancy in years	62.35	72.02	70.79	64.27‡	65.61
GDP per capita in US$	$556.81	$4603.42	$1901.25	$1444.46§	$1194.12
2015–20 inflation multiplier based on GDP deflator	2.15	1.12	1.21	1.47	1.28

Parameters shown were implemented in the model as point estimates. The cost, life expectancy, and inflation multipliers did not vary across the 10000 runs for country-specific results. These estimates were largely used to model key features of each country. Health-care use costs were derived from previously published results,^7^ and inflated to 2020 US$ using the GDP price deflator in each country from World Bank data, except for costs for India which were estimated by local staff at the Belagavi, India site and reflect 2021 private hospital data. Life expectancy data are from the WHO Global Health Observatory, 2019 for both sexes combined. CPAP=continuous positive airway pressure. GDP=gross domestic product. NA=non-applicable.

* The larger cost of the two countries was selected if the costs were different.

† Oxygen, CPAP, and mechanical ventilation were not administered at the site in DR Congo.

‡ Mean of life expectancy in Kenya was 66⋅09 years and 62⋅45 years in Zambia.

§ Mean of GDP per capita in Kenya was $1838 and $1051 in Zambia.

**Table 3:** Cost-effectiveness of low-dose aspirin treatment for nulliparous, singleton pregnancies expressed per 10000 pregnancies

	DR Congo	Guatemala	India	Kenya and Zambia*	Pakistan

DALYs averted, years	2155 (1267–2787)	2251 (1324–2912)	2241 (1318–2899)	2176 (1280–2815)	2190 (1288–2833)
Cost-effectiveness outcomes					
Intervention cost, US$	$64 517.21 (43 966.78–84 904.91)	$33 609.31 (22 904.19–44 230.01)	$36 309.45 (24 743.92–47 783.78)	$44 111.36 (30 060.59–58 050.60)	$38 470.02 (26216.49–50627.23)
Hospitalisation costs averted, US$	$1772.34 (129.38–2238.83)	$4158.72 (3381.04–4890.17)	$4552.80 (3799.01–5181.32)	$3887.38 (3072.76–4673.09)	$3545.15 (2804.35–4252.86)
Incremental cost, US$	$62 745.42 (45 903.29–79 289.01)	$29 450.03 (23 940.67–34 632.10)	$31 758.22 (26 513.04–36 154.77)	$40 225.18 (31 797.48–48 363.67)	$34 925.93 (27621.48–41897.50)
Cost per preterm birth averted, US$	$445.10 (338.17–730.98)	$208.81 (159.43–343.33)	$225.05 (171.19–369.58)	$286.33 (216.45–468.96)	$248.96 (187.97–407.16)
Cost per perinatal death averted, US$	$847.13 (654.33–1439.02)	$397.48 (306.79–675.25)	$429.14 (331.44–728.57)	$543.44 (419.26–922.80)	$471.32 (364.18–801.30)
Cost per DALY averted, US$	$29.12 (22.51–49.51)	$13.08 (10.11–22.24)	$14.17 (10.96–24.10)	$18.48 (14.29–31.43)	$15.95 (12.33–27.11)
Cost per DALY averted as a proportion of gross domestic product per capita	5.2% (4.0–8.9)	0.3% (0.2–0.5)	0.7% (0.6–1.3)	1.3% (1.0–2.2)	1.3% (1.0–2.3)

Data are mean (95% CI) of 10000 model simulations, each using a different parameter set (drawn from the same distributions). Model results for preterm births averted, perinatal deaths averted, hospitalisations averted, and intervention cost are based on the point estimates in table 1. The mean result from the 10000 simulations might vary slightly from the point estimates in table 1. Country-specific analyses reflect health-care use costs and life expectancy data from that country or group of countries in table 2. DALY=disability-adjusted life year.

* Results based on the mean life expectancy and health-care use costs for Kenya and Zambia.

**Table 4:** One-way sensitivity analyses on the cost and effectiveness of LDA expressed per 10000 pregnancies

	Base case LDA cost and effectiveness (Pakistan)	Low LDA cost and effectiveness	High LDA cost and effectiveness

LDA regimen cost,* US$	$3.83	$0.92	$8.54
Parameters adjusted			
Intervention cost, US$	$38 470.02 (26 216.49–50 627.23)	$9230.40 (6290.31–12 147.37)	$85 381.20 (58 185.45–112 363.17)
Cost-effectiveness outcomes, US$			
Incremental cost	$34 924.93 (27 621.48–41 897.50)	$5685.31 (4496.41–6820.35)	$81 775.29 (64674.56–8101.30)
Cost per preterm birth averted	$247.96 (187.97–407.16)	$40.36 (30.60–66.28)	$568.24 (933.09–430.77)
Cost per perinatal death averted	$471.32 (364.18–801.30)	$76.72 (59.28–130.40)	$1090.34 (842.48–1853.07)
Cost per DALY averted	$15.95 (12.33–27.11)	$2.60 (2.01–4.41)	$36.89 (28.51–62.72)
LDA effectiveness in reducing preterm birth,† relative risk	0.89	0.98	0.81
Parameters adjusted			
Preterm births averted	140.85 (85.78–185.80)	26.17 (24.45–27.90)	248.57 (232.23–265.06)
DALYs averted	2190.37 (1288.41–2833.47)	2190.37 (1288.41–2833.47)	2190.37 (1288.41–2833.47)
Cost-effectiveness outcomes, US$			
Incremental cost	..	$36 473.79 (29 305.97–43 402.61)	$33 469.44 (26 401.51–40 172.20)
Cost per preterm birth averted	..	$1393.98 (1307.25–1492.05)	$134.65 (126.27–144.12)
Cost per perinatal death averted	..	$492.22 (380.33–836.55)	$451.68 (349.00–767.65)
Cost per DALY averted	..	$16.65 (12.87–28.32)	$15.28 (11.81–25.98)
LDA effectiveness in reducing perinatal death‡, relative risk	0.86	1.00§	0.74
Parameters adjusted			
Perinatal deaths averted	74.10 (43.60–95.90)	1.60 (1.40–1.80)	139.40 (124.30–154.70)
DALYs averted	2190.37 (1288.41–2833.47)	47.68 (42.61–52.84)	4117.10 (3672.89–4569.68)
Cost-effectiveness outcomes, US$			
Incremental cost	..	$36 532.09 (29 242.96–43 515.22)	$33 481.29 (26 370.93–40 229.07)
Cost per preterm birth averted	..	$259.37 (425.90–196.62)	$237.71 (180.20–390.33)
Cost per perinatal death averted	..	$22 832.56 (20 295.61–26 094.35)	$240.18 (216.43–296.36)
Cost per DALY averted	..	$766.14 (691.43–857.27)	$8.13 (7.33–9.12)

These data are clinical and cost-effectiveness results from our sensitivity analyses. They represent the mean of 10000 runs of the model for each reported outcome. Results reflect health-care use cost and life expectancy data from the median country (Pakistan), which represented the median life expectancy, GDP per capita, and cost-effectiveness results among the ASPIRIN trial sites. DALY=disability-adjusted life year. GDP=gross domestic product. LDA=low-dose aspirin. RR=relative risk.

* The LDA cost represents the cost of the entire 217-day supply of 81 mg once-a-day LDA tablets in Pakistan; low-cost and high-cost estimates were based on the low ($0⋅0042 per day) and the high ($0⋅0393 per day) price per tablet in the 2015 International Medical Products Price Guide inflated to 2020 using the GDP price deflator from the World Bank for Pakistan.

† Low and high LDA effectiveness estimates were based on the upper and lower 95% CI reported in the ASPIRIN trial;^4^ preterm births averted reflect the estimate from the analyses in table 3; DALYs averted reflect the estimate from Pakistan (table 3).

‡ Low and high LDA effectiveness estimates were based on the upper and lower 95% CI reported in the ASPIRIN trial;^4^ perinatal deaths averted reflect the estimate from the analyses in table 3; DALYs averted reflect the estimate from Pakistan (table 3).

§ The rounded RR of perinatal death is shown (1⋅00); the actual upper bound of the 95% CI of the RR used to reflect ASPIRIN trial findings was 0⋅997.

## Data Availability

Deidentified participant data from the ASPIRIN trial are available at the National Institute of Child Health and Human Development repository. Data sharing governed according to the procedures and policies of N-Dash. A data dictionary is also provided.

## Research in context

### Evidence before this study

In 2020, the ASPIRIN trial, done by the National Institute of Child Health and Human Development Global Network for Women’s and Children’s Health Research, showed the efficacy of low-dose aspirin (LDA) initiated in the first trimester for nulliparous, singleton pregnancies to prevent preterm birth in low-income and middle-income countries. We searched PubMed from the inception of the database to Oct 14, 2022, for manuscripts on the cost-effectiveness of aspirin in pregnancy for improved pregnancy outcomes without language restrictions using search terms “aspirin” AND “cost-effectiveness” AND (“pregnancy” OR “preterm birth” OR “preeclampsia”). We found 34 articles, of which eight were cost-effectiveness analyses of aspirin. These eight studies evaluated the cost-effectiveness of aspirin in the prevention of pre-eclampsia, and all were focused on costs relevant to high-income countries.

### Added value of this study

Our study shows the cost-effectiveness of LDA for nulliparous, singleton pregnancies for the prevention of preterm birth in low-income and middle-income countries. This analysis explores the cost-effectiveness of LDA in each country represented in the ASPIRIN trial using country-specific costs.

### Implications of all the available evidence

LDA treatment in nulliparous, singleton pregnancies is a low-cost, effective treatment to reduce preterm birth as well as perinatal death. Given the estimated cost per disability-adjusted life year averted, policy makers should prioritise this intervention for implementation in publicly funded health care in low-income and middle-income countries.
