# Supplementary material for: Cost-effectiveness of low-dose aspirin for the prevention of preterm birth: a prospective study of the Global Network for Women’s and Children’s Health Research
Source: Lancet Glob Health. Author manuscript; Available in PMC 2023 Jun 23. (PMC10288322; doi:10.1016/S2214-109X(22)00548-4)
Supplement: MMC1 [file NIHMS1883714-supplement-MMC1.pdf]

THE LANCET
Global Health

Supplementary appendix

This appendix formed part of the original submission and has been peer reviewed. We post it as supplied by the authors.

Supplement to: Patterson JK, Neuwahl S, Goco N, et al. Cost-effectiveness of low-dose aspirin for the prevention of preterm birth: a prospective study of the Global Network for Women's and Children's Health Research. *Lancet Glob Health* 2023; **11:** e436–44.

Appendix

Table of Contents

Supplemental Table 1. Institutional review board approvals for the ASPIRIN trial……………………………………2

Supplemental Table 2. Members of the ASPIRIN Study Group of the
Global Network for Women's and Children's Health Research…………………………………………………...3

***Supplemental Table 1.* Institutional review board approvals for the ASPIRIN trial**

**Institutional Review Board**	**Reference No.**
Thomas Jefferson University, Philadelphia, Pennsylvania	Control #16F.320
JN Medical College (JNMC), Belagavi (Belgaum), India	MDC/IECHSR/2016-17/A-72
University of North Carolina at Chapel Hill, Chapel Hill, North Carolina	No. 15-1780
Kinshasa School of Public Health (KSPH), Kinshasa, Democratic Republic of Congo	ESP/CE/082/2015
University of Alabama, Birmingham, Alabama	F150611007
University of Zambia, Lusaka, Zambia	REF No. 007-08-15
University of Colorado Denver Anschutz Medical Campus, Aurora, Colorado	COMIRB Protocol 15-1625
Institute for Nutrition in Central America and Panama (INCAP), Guatemala City, Guatemala, (Universidad Francisco Marroquín)	CE-FM/UFM-055-15
Columbia University, New York City, New York	IRB-AAAP5506
Aga Khan University (AKU), Karachi, Pakistan	3562-CHS-ERC-15
Boston University, Boston, Massachusetts	H-35466
Lata Medical Research Foundation, Nagpur, India	RPC #21
Indiana University School of Medicine, Indianapolis, Indiana	1507246903R001
Moi University, Eldoret, Kenya	IREC/2015/81 Approval No. 0001429
RTI International, Research Triangle Park, North Carolina	12940-7

Supplemental Table 2. **Members of the ASPIRIN Study Group of the Global Network for Women's and Children's Health Research**

First Name	Surname	Institution	Country
Jean	Okitawutshu	Kinshasa School of Public Health	Democratic Republic of Congo
Adrien	Lokangaka	Kinshasa School of Public Health	Democratic Republic of Congo
Antoinette	Tshefu	Kinshasa School of Public Health	Democratic Republic of Congo
Javier	Chicuy	Instituto de Nutrición de Centro América y Panamá (INCAP)	Guatemala
Lester	Figueroa	Instituto de Nutrición de Centro América y Panamá (INCAP)	Guatemala
Ana	Garces	Instituto de Nutrición de Centro América y Panamá (INCAP)	Guatemala
Umesh S.	Charantimath	KLE Academy of Higher Education and Research J N Medical College, Belgavi	India
Madiwalayya S.	Ganachari	KLE Academy of Higher Education and Research J N Medical College, Belgavi	India
Shivaprasad S.	Goudar	KLE Academy of Higher Education and Research J N Medical College, Belgavi	India
Narayan V.	Honnungar	KLE Academy of Higher Education and Research J N Medical College, Belgavi	India
Avinash	Kavi	KLE Academy of Higher Education and Research J N Medical College, Belgavi	India
Bhalchandra S.	Kodkany	KLE Academy of Higher Education and Research J N Medical College, Belgavi	India
Mrityunjaya C.	Metgud	KLE Academy of Higher Education and Research J N Medical College, Belgavi	India
Yogesh S.	Kumar	KLE Academy of Higher Education and Research J N Medical College, Belgavi	India
Manjunath S.	Somannavar	KLE Academy of Higher Education and Research J N Medical College, Belgavi	India
Sunil S.	Vernekar	KLE Academy of Higher Education and Research J N Medical College, Belgavi	India
Geetanjali M.	Katageri	S.N. Medical College, Bagalkot	India
Ashalata A.	Mallapur	S.N. Medical College, Bagalkot	India
Umesh Y.	Ramadurg	S.N. Medical College, Bagalkot	India
Prabir	Das	Lata Medical Research Foundation	India
Kunal	Kurhe	Lata Medical Research Foundation	India
Archana	Patel	Lata Medical Research Foundation	India
Emmah	Achieng	Department of Child Health and Paediatrics, Moi University School of Medicine	Kenya
Paul	Nyongesa	Department of Child Health and Paediatrics, Moi University School of Medicine	Kenya
Fabian	Esamai	Department of Child Health and Paediatrics, Moi University School of Medicine	Kenya
Saleem	Jessani	Aga Khan University	Pakistan
Sarah	Saleem	Aga Khan University	Pakistan
Farnaz	Naqvi	Aga Khan University	Pakistan
Zahid	Soomro	Aga Khan University	Pakistan
Seemab	Naqvi	Aga Khan University	Pakistan
Musaku	Mwenechanya	University Teaching Hospital	Zambia
Elwyn	Chomba	University Teaching Hospital	Zambia
Waldemar A.	Carlo	University of Alabama at Birmingham	United States
Patricia L.	Hibberd	Boston University School of Public Health	United States
Nancy F.	Krebs	University of Colorado	United States
Robert L.	Goldenberg	Columbia University	United States
Matthew	Hoffman	Indiana University	United States
Christina	Care	Indiana University	United States
Edward	Liechty	Indiana University	United States
Sherri	Bucher	Indiana University	United States
Melissa	Bauserman	University of North Carolina at Chapel Hill	United States

Carl	Bose	University of North Carolina at Chapel Hill	United States
Richard	Derman	Thomas Jefferson University	United States
Robert	Silver	University of Utah	United States
Robert	Nathan	University of Washington	United States
Marion	Koso-Thomas	Eunice Kennedy Shriver National Institute of Child Health and Human Development	United States
Menachem	Miodovnik	Eunice Kennedy Shriver National Institute of Child Health and Human Development	United States
Elizabeth M.	McClure	RTI International	United States
Dennis	Wallace	RTI International	United States
Suchita	Parepalli	RTI International	United States
Tracy	Nolen	RTI International	United States
Janet	Moore	RTI International	United States
Elizabeth	MacGuire	RTI International	United States
Norman	Goco	RTI International	United States
Jennifer	Hemingway-Foday	RTI International	United States
